# The impact of the implementation of physician assistants in inpatient care: A multicenter matched-controlled study

**DOI:** 10.1371/journal.pone.0178212

**Published:** 2017-08-09

**Authors:** Marijke J. C. Timmermans, Anneke J. A. H. van Vught, Yvonne A. S. Peters, Geert Meermans, Joseph G. M. Peute, Cornelis. T. Postma, P. Casper Smit, Emiel Verdaasdonk, Tammo S. de Vries Reilingh, Michel Wensing, Miranda G. H. Laurant

**Affiliations:** 1 Radboud Institute for Health Sciences, Scientific Center for Quality of Healthcare (IQ healthcare), Radboud university medical center, Nijmegen, The Netherlands; 2 Faculty of Health and Social Studies, HAN University of Applied Sciences, Nijmegen, The Netherlands; 3 Department of Orthopaedics, Bravis Hospital, Bergen op Zoom and Roosendaal, The Netherlands; 4 Department of Cardiology, VieCuri Medical Center Noord-Limburg, Venlo, The Netherlands; 5 Department of Internal Medicine, Radboud university Nijmegen medical center, Nijmegen, The Netherlands; 6 Department of Surgery, Reinier Haga Groep, Delft, The Netherlands; 7 Department of Surgery, Jeroen Bosch Hospital, ‘s-Hertogenbosch, The Netherlands; 8 Department of Surgery, Elkerliek Hospital, Helmond, The Netherlands; 9 Department of General Practice and Health Services Research, Heidelberg University Hospital, Heidelberg, Germany; University of Colorado Denver, UNITED STATES

## Abstract

**Background:**

Medical care for admitted patients in hospitals is increasingly reallocated to physician assistants (PAs). There is limited evidence about the consequences for the quality and safety of care. This study aimed to determine the effects of substitution of inpatient care from medical doctors (MDs) to PAs on patients’ length of stay (LOS), quality and safety of care, and patient experiences with the provided care.

**Methods:**

In a multicenter matched-controlled study, the traditional model in which only MDs are employed for inpatient care (MD model) was compared with a mixed model in which besides MDs also PAs are employed (PA/MD model). Thirty-four wards were recruited across the Netherlands. Patients were followed from admission till one month after discharge. Primary outcome measure was patients’ LOS. Secondary outcomes concerned eleven indicators for quality and safety of inpatient care and patients’ experiences with the provided care.

**Results:**

Data on 2,307 patients from 34 hospital wards was available. The involvement of PAs was not significantly associated with LOS (β 1.20, 95%CI 0.99–1.40, *p* = .062). None of the indicators for quality and safety of care were different between study arms. However, the involvement of PAs was associated with better experiences of patients (β 0.49, 95% CI 0.22–0.76, *p* = .001).

**Conclusions:**

This study did not find differences regarding LOS and quality of care between wards on which PAs, in collaboration with MDs, provided medical care for the admitted patients, and wards on which only MDs provided medical care. Employing PAs seems to be safe and seems to lead to better patient experiences.

**Trial registration:**

ClinicalTrials.gov Identifier: NCT01835444

## Background

Medical care for admitted patients is increasingly reallocated to physician assistants (PAs), because of an increased appreciation of continuity of care, pressure to deliver healthcare efficiently, and local shortages of medical doctors (MDs) [1–3]. A PA is a non-physician healthcare professional licensed to practice medicine in defined domains, with variable degrees of professional autonomy [4]. PAs who are employed for medical care for admitted patients usually work in a team compromising both PAs and MDs (i.e. residents, staff physicians or hospitalists). Although there is a worldwide trend of an increase of PAs in the management of hospitalized patients, evidence about the consequences of reallocating inpatient care from MDs to PAs for healthcare outcomes is limited.

Literature suggests that PAs add to the quality and safety of care, which may overall reduce patients’ length of stay in hospitals [1]. The turnover of house staff is traditionally high due to use of recent medical graduates who are planning to do fellowships and the mandatory rotational cycles. PAs generally do not rotate and thus enhance continuity of care. Increased provider continuity has been associated with improved patient outcomes and more positive evaluations of medical care by patients [5, 6]. However, most of these continuity of care studies did not focus on inpatient care.

Several North-American studies showed that quality and efficiency of care provided by PAs is similar to that of MDs, with high levels of patient satisfaction [7, 8]. However, the majority of these studies focused on primary care or intensive care units only. Only a few studies have compared non-acute inpatient care delivered by a PA-based team with the care delivered by a resident-based team [9–13]. These studies suggested similar quality of care, but results of PA employment on length of stay (LOS) varied across the studies. All studies concerned only one clinical discipline and thus a limited variation of patients. Given the outcomes of these studies and their limitations, we conducted a multicenter study that included PAs providing care to hospitalized patients including different clinical disciplines and hospitals.

### Study aim

This study aimed to determine the effects of substitution of inpatient care from MDs to PAs on patients’ LOS, quality and safety of care, and patient experiences. We hypothesized that medical care by PAs is, compared to MDs, more standardized and more continued, which will be reflected by shorter hospital stay. Secondary hypothesis is that medical care by PAs results in at least as good quality and safety of care and better patient experiences.

## Methods

### Study design and population

A multicenter, non-randomized, matched-controlled study was performed in the Netherlands, comparing wards with a mixed ‘PA/MD model’ (intervention group) with wards with a solely ‘MD model’ (control group). The study design has been described in detail elsewhere [14, 15]. In summary, control wards were matched with the intervention wards on the basis of medical specialty and hospital type (i.e. academic versus non-academic). Hospital wards were assigned to the intervention group if the PA covered at least 51% of the available ward care hours per week during dayshifts (8 a.m. till 18 p.m.) on weekdays. Wards were assigned to the control group if exclusively MDs provided medical care.

### Description of the models for the organization of medical care at the ward

#### MD model

In the MD model, only MDs are in charge of the admitted patients at a specific hospital department. Most of them are residents [15]. The resident is physically present at the department for at least a couple of hours each weekday, and is the first point of access to medical care during office hours. Their work includes daily clinical care and patient management. The residents are supervised by attending physicians. In some cases, especially in smaller hospitals where often no residents are employed, the medical specialists provide all medical care for the admitted patients.

#### PA/MD model

In the PA/MD model, the PAs who were employed at the wards are substitutes for the residents. Their tasks and responsibilities are largely comparable. PAs have the same authorizations as residents: they can make indications for treatment, perform predefined medical procedures and subscribe medication independently [16]. In a previous publication we described the characteristics and tasks of all PAs and residents who were primary employed for medical ward care [15]. Although PAs had comparable core tasks as residents, the amount of time which was spent per group of tasks slightly varied across the professionals. PAs spent relatively more time on direct inpatient care, while the MDs spent relatively more time on additional tasks like outpatient contacts and medical procedures.

We included two different models within the intervention group (PA/MD model): a model in which PAs collaborate with residents and a model in which only PAs are the first point of access to medical care. In both models, the PAs as well as the residents were supervised by attending physicians. As described previously, median 68% (IQR 48–77) of the hours for medical care at the ward were covered by PAs [15].

### Study population

The focus of this study was on the patients admitted to the hospital wards. Exclusion criteria for patients were: 1) Younger than 18 years; 2) Terminally ill; and 3) Receiving daycare. Daycare was defined as hospital admissions that were (according to hospital protocols) intended to last 24 hours or less.

### Outcome measures

#### Length of stay

Length of stay (LOS) was the primary outcome measure of the study. We defined LOS as the time in days between the dates of discharge and admission. Both dates were derived from patient medical records by trained medical students and researchers. As often the involved PA or resident informed the medical student or researcher about which data could be found where in the patient records, it was not possible to blind the assessors. To minimize information bias, a random sample of 10% of the patient records per ward was analyzed by a second researcher, who was blinded for the outcome of the initial researcher. In case of an inter-rater agreement of less than 95%, the records of the total sample were reassessed.

#### Quality and safety of care

A set of clinical indicators and process indicators was composed to measure the quality and safety of medical care at the ward. First, 20 provisional indicators were identified from scientific literature and from existing indicators, such as the national set of indicators for quality of hospital care from the Dutch Health Care Inspectorate (IGZ) [17]. This selection was based on potential relevance for a diversity of medical specialties. Second, the relevance and feasibility of the provisional set of indicators was discussed with an expert panel of physicians. Finally, a set of eleven clinical and process indicators was selected (Table 1). All indicators covered the admission period till a maximum of one month after discharge. Data were retrospectively derived from patient medical records and patient questionnaires. We randomly reassessed 10% of all patient records per ward to increase internal validity.

**Table 1:** Clinical and process indicators for quality and safety of medical care.

**Clinical indicators**
• In-hospital mortality
• Unplanned transfer to intensive care unit
• Cardiopulmonary resuscitation
• Pressure ulcer developed during admission
• Fever: incidence of episodes of two days that body temperature ≥38
• Pain score: incidence of episodes of two days that had a Numeric Rating Score ≥7
• Hospital infections: infusion-, urinary track-, airway-, and postoperative wound infections
• Presentation at department of emergency, within one month after discharge
• Non-elective readmission within one month after discharge
**Process indicators**
• Days between discharge and letter of discharge
• Introduction by the PA or MD to the patient within 24 hours after hospital admission

Abbreviations: PA = physician assistant; MD = medical doctor

#### Patient experiences with medical ward care

Patient experiences with medical care were assessed by a self-administered questionnaire at discharge. This questionnaire focused on satisfaction with communication, experienced continuity of care and cooperation between care providers, and the patients view on the medical competencies of the PA or MD. Patient perceptions on communication skills were measured with the Communication Assessment Tool (CAT), a validated questionnaire which consists 14 questions which can be rated on a five point Likert scale, ranging from ‘poor’ to excellent’[18]. The Cronbachs’ alpha in our study was 0.98. Three subscales from the validated ‘Chronically Ill Patients Evaluate general Practice’ (CEP) questionnaire [19] were added to measure the items satisfaction with continuity of care (one question), cooperation of ward care providers (one question), and medical care (three questions) (Cronbachs’ alpha 0.93). Each item was rated on a six point Likert scale, ranging from ‘poor’ to ‘excellent’. Patients could additionally score an item as ‘not applicable’. At last, one question was added about general satisfaction with medical care at the ward (scale 1–10). To ensure that patients knew who their attending PA or MD was, we included photos from the medical care provider(s) in the questionnaire.

### Sample size calculation

The originally published sample size calculation [14] was adjusted prior to start of data collection [20]. To detect a relative difference in LOS of 20% between the ‘PA/MD model’ and ‘MD model’, assuming an average LOS of 6 days (SD 4.9), alpha 5%, power 80% and an intra cluster coefficient of 0.06 for patients in same ward, 30 wards including 100 patients each were required. Taking into account an expected drop-out of a maximum of two matched pairs, 34 wards (17 in each arm) with each 100 patients were required. In case of no drop-out, 50 patients per ward would be sufficient.

### Data analyses

Differences at baseline between groups were analyzed using the χ^2^ test, *t* test, or Fisher exact test. To compare intervention wards with control wards, we used linear regression analyses for continuous outcomes (LOS, patient experiences). Because of the non-normal distribution of LOS (skewed to the higher scores) data were log-transformed before analysis. For each domain of patient experiences (i.e. communication, continuity of care, cooperation, medical competencies) an average score was calculated per patient for further analyses. In case more than 75% of the answers were missing, no aggregated score was calculated. Logistic regression analysis was conducted for the dichotomous outcomes (i.e. indicators for quality and safety of care). Random coefficients were added to all regression models to account for statistical clustering of data in hospital wards. All analyses were done on an intention-to-treat basis. Matching was taken into account by adding covariables for the matching variables (i.e hospital type and medical specialty).

Multivariable models were constructed to adjust for potential confounders (Table 2). Covariables were included in the final model only if they modified the regression coefficient of the central determinant by more than 10%, regardless of statistical significance of effects. In all analyses, two-tailed p-values of 0.05 or lower were considered statistically significant. All presented estimates were adjusted for the matched design, either without or with correction for confounding. To explore heterogeneity within the results, post-hoc subgroup analyses were performed for each submodel of medical ward care, i.e. 1) the MS model: medical specialists are in charge of all admitted patients; 2) MR model: residents or junior doctors are in charge of all admitted patients; 3) mixed PA/MR model: both residents and PAs are in charge of the admitted patients; 4) PA model: PAs are in charge of all admitted patients [15]. We also conducted separate analyses for the surgical specialties (i.e. surgery, orthopaedics, head and neck oncology surgery) and the non-surgical specialties (i.e. gastroenterology, pulmonology, cardiology).

**Table 2:** Baseline characteristics of patients.

Baseline characteristic	PA/MD model (n = 1021)	MD model (n = 1286)	*P* Value
Medical specialty *n(%)*			< .001
Surgery	601 (59%)	696 (54%)	
Gastroenterology	102 (10%)	181 (14%)	
Pulmonology	91 (9%)	107 (8%)	
Cardiology	101 (10%)	124 (10%)	
Orthopaedics	103 (10%)	100 (8%)	
ENT, head and neck oncology surgery	23 (2%)	78 (6%)	
Hospital type *n(%)*			< .001
Teaching	552 (55%)	709 (53%)	
Academic	23 (2%)	78 (3%)	
Non-academic	529 (52%)	631 (50%)	
Non-teaching	469 (56%)	577 (57%)	
Gender, male *n(%)*	524 (53%)	682 (54%)	.47
Age, years *mean ± SD*	64 ± 16	63 ± 15	.11
Major diagnoses *n(%)*			< .001
Digestive system	204 (20%)	247 (19%)	
Circulatory system	158 (16%)	274 (22%)	
Neoplasms	108 (11%)	195 (15%)	
Musculoskeletal system and connective tissue	120 (12%)	119 (9%)	
Injury and poisoning	135 (13%)	80 (6%)	
Infectious and parasitic diseases	59 (6%)	81 (6%)	
Respiratory system	51 (5%)	75 (6%)	
Symptoms	61 (6%)	87 (7%)	
Charlson index for co-morbidity score *mean ± SD (% with score ≥1)*	1.1 *±* 1.8 (43%)	1.1 *±* 1.8 (44%)	.65 .66
Highest education *n(%)*			.15
Low	371 (38%)	422 (34%)	
Middle	380 (39%)	489 (40%)	
High	233 (24%)	328 (27%)	
Ethnicity, Dutch *n(%)*	976(99%)	1212 (98%)	.15
Marital status *n(%)*			.29
No partner	136 (14%)	167 (14%)	
Partner	730 (74%)	949 (77%)	
Widow	119 (12%)	125 (10%)	
Smoking status *n(%)*			.65
No, never smoked	325 (33%)	385 (31%)	
No, but ever smoked	494 (48%)	626 (50%)	
Yes, still smoking	174 (17%)	230 (19%)	
Body Mass Index *(mean ± SD)*	27 *±* 5	27 *±* 5	.79
Number of hospitalizations for same problem *n(%)*			.20
1 hospitalization	580 (59%)	693 (56%)	
>1 hospitalization	403 (41%)	540 (44%)	
Type of admission *n(%)*			< .001
Elective	402 (41%)	687 (56%)	
Urgent	588 (59%)	547 (44%)	
Discharge destination *n(%)*			< .001
Home	765 (90%)	965 (92%)	
Hospital	12 (1%)	30 (3%)	
Nursing home/rehabilitation center/hospice	56 (7%)	28 (3%)	
Family relative	18 (2%)	25 (2%)	
Health related quality of life at admission	63 *±* 19	64 *±* 20	.08
Workload at the ward: minutes per bed per week *(mean ± SD)*	111 *±* 48	130 *±* 72	< .001

Numbers may not add up to the total because of missing values. Abbreviations: PA/MD model = Both PAs and MDs (i.e. medical specialists or residents) are in charge of the admitted patients. MD model = Only MDs are in charge of the admitted patients

### Ethical considerations

The Research Ethics Committee of the Radboud University Medical Center Nijmegen waived the need for ethics approval (registration number: 2012/306). All data were handled strictly confidential and written informed consent was obtained from all patients.

## Results

**Fig 1:**
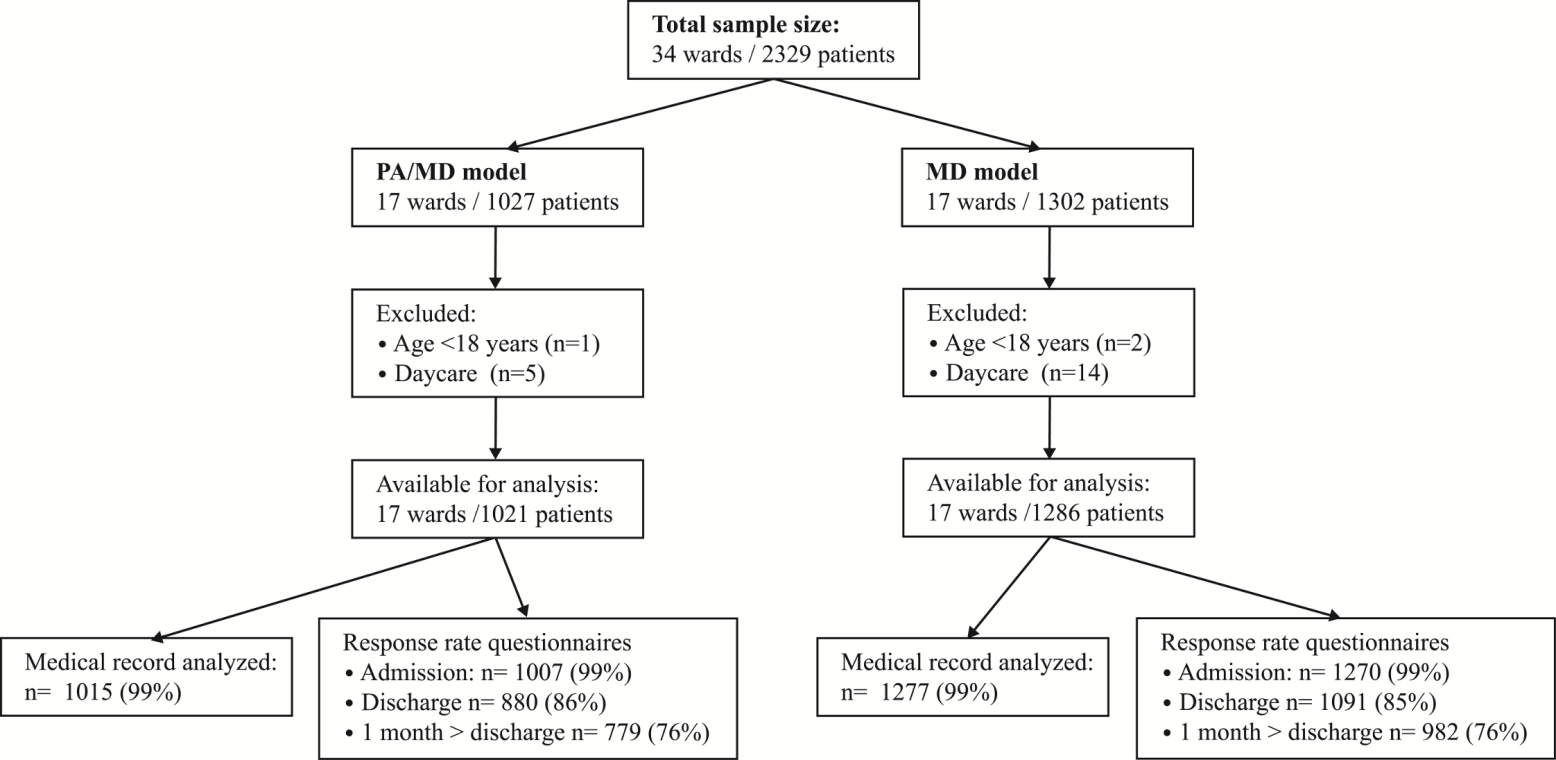
Flow-chart of patients.

We included 1,021 patients spread over 17 hospital wards in the intervention group (PA/MD model), and 1,286 patients spread over 17 hospital wards in the control group (MD model) (Fig 1). The main patient characteristics are summarized in Table 2. Most characteristics were well balanced between the two groups. More patients in the intervention group were acutely admitted (59% versus 44% in the control group, p< .001). Also the primary diagnosis differed significantly.

### Length of stay

We had complete data about LOS of 99% of the patients (Fig 1). Results for the crude and adjusted associations between the organizational models and LOS are shown in Table 3. Median LOS of the patients in the intervention group was 6 days (IQR 4–10), median LOS of the patients in the control group was 5 days (IQR 4–8). The involvement of PAs was not significantly associated with the crude LOS (β 1.22, 95% CI 0.99–1.51, *p* = .062). The beta of the final model did not change substantially after adjustment for potential confounders and remained non-significant (β 1.20, 95% CI 0.99–1.40, *p =* .064).

**Table 3:** Length of hospital stay and indicators for quality and safety of care.

Outcome	PA/MD model	MD model	Crude^c^		Adjusted^d^	
			B	95% CI	β	95% CI
**Length of hospital stay** *median (IQR)* ^a^	6 (4–10)	5 (4–8)	1.22	0.99–1.51	1.20	0.99–1.40
**Indicators for quality and safety of care**			**OR**	**95% CI**	**OR**	**95% CI**
In-hospital mortality *n(%)*	2/1021 (0.2%)	1/1285 (0.1%)	NA	NA	NA	NA
Unplanned transfer to ICU *n(%)*	19/987 (2%)	23/1242 (2%)	0.92	0.48–1.76	1.08	0.68–1.71
Cardiopulmonary resuscitation *n(%)*	1/988 (0.1%)	1/1228 (0.1%)	NA	NA	NA	NA
Pressure ulcer developed during admission *n(%)*	31/889 (4%)	10/1116 (1%)	0.72	0.46–1.14	0.70	0.43–1.13
Episode of at least 2 days temp ≥38 *n(%)*	94/974 (10%)	120/1230(10%)	0.90	0.67–1.21	0.92	0.67–1.26
Episode of at least 2 days pain score ≥7 *n(%)*	57/978 (6%)	34/1165(3%)	1.60	1.09–2.35**	1.55	0.97–2.48
Hospital infection^b^ *n(%)*	62/980 (6%)	65/1212 (5%)	0.97	0.67–1.42	1.12	0.75–1.68
Presentation at department of emergency *n(%)*	119/743 (16%)	169/941 (18%)	0.83	0.64–1.08	0.79	0.60–1.05
Unplanned readmission *n(%)*	66/738 (9%)	77/935 (8%)	1.09	0.76–1.55	1.10	0.75–1.62
Introduction to patient <24h *n(%)*	658/960 (69%)	820/1190 (69%)	0.88	0.68–1.14	0.81	0.62–1.06
**Indicators for quality of care**			**B**	**95% CI**	**β**	**95% CI**
Days between discharge and discharge letter *median (IQR)* ^a^	1 (0–7)	4 (0–14)	-0.18	-0.80–0.44	-0.19	-0.81–0.43

Abbreviations: NA = not applicable because of limited number of cases; IQR = interquartile range; PA/MD model = Both PAs and MDs (i.e. medical specialists or residents) are in charge of the admitted patients. MD model = Only MDs are in charge of the admitted patients.

a. log-transformed before regression analysis

b. including Infusion, urinary track, airway and/or postoperative wound infection

c. Adjusted for match criteria medical specialty and hospital type

d. Adjusted for match criteria and the confounders primary diagnosis, type of admission and discharge destination

** *P* < .05

### Quality and safety of care

We were able to check 99% of all patient records. Item-missing varied from 1% (in-hospital mortality) to 24% (discharge letter). Incidence of unplanned readmission and presentation at the emergency department were derived from the patient questionnaire, which was sent one month after discharge. The response rate on this questionnaire was 76% in both study arms (Fig 1). The indicator ‘incidence of episode of at least two days pain score ≥ 7’ showed a significant association with the inpatient care model (OR 1.60, 95% CI 1.09–2.35) when not adjusted for confounding. After adjustment for confounders, none of the indicators for quality and safety of inpatient care were related to the involvement of PAs (Table 3).

### Patient experiences

The response rate on the questionnaire at discharge was 86% in the intervention group and 85% in the control group (Fig 1). The item non-response rate varied from 15% to 27%, including the questions answered with ‘not applicable’. The overall evaluation of medical care by patients was on average 8.4 ± 1.3 in the intervention group and 8.0 ± 1.5 in the control group. The involvement of PAs was significantly associated with more positive overall evaluations of care by patients (β 0.49, 95% CI 0.22–0.76, *p* = .001). Experiences of patients with all separate domains communication, continuity, cooperation and medical care were also significantly better on the wards that involved PAs (Table 4).

**Table 4:** Patient experiences with care.

Outcome	PA/MD model (n = 849)	MD model (n = 1001)	Crude^a^		Adjusted^b^	
	Mean (SD)	Mean (SD)	β	95% CI	β	95% CI
Overall evaluation score	8.4 (1.3)	8.0 (1.5)	0.48**	0.21–0.74	0.49**	0.22–0.76
Communication (15 items)	4.2 (0.7)	4.0 (0.8)	0.24**	0.09–0.38	0.25**	0.09–0.40
Continuity (1 item)	4.7 (1.1)	4.4 (1.2)	0.35**	0.13–0.57	0.32**	0.10–0.55
Cooperation (1 item)	4.7 (1.1)	4.4 (1.2)	0.33**	0.10–0.56	0.31**	0.09–0.54
Medical care (2 items)	4.8 (1.0)	4.7 (4.0)	0.28**	0.04–0.51	0.28**	0.05–0.52

Higher scores reflect better evaluation of care. Communication was measured on a 5 point likert scale; continuity, cooperation and medical care on a 6 point likert scale. Overall satisfaction on a 1–10 scale. Abbreviations: PA/MD model = Both PAs and MDs (i.e. medical specialists or residents) are in charge of the admitted patients. MD model = Only MDs are in charge of the admitted patients

a. Adjusted for match criteria medical specialty and hospital type

b. Adjusted for match criteria and the confounders primary diagnosis, type of admission and discharge destination

** *P* < .05

### Subgroup analyses

Results for the analyses per submodel of medical ward care are shown in S1 Table. No differences were found between the groups for LOS. Regarding the indicators for quality and safety, we found significant differences for the incidence of hospital infections, pressure ulcer, episode of two days body temperature ≥38, and episodes of two days Numeric Rating Score ≥7. The scores on these indicators were lowest for the MS model. Patient evaluations were significantly highest for the PA model and the mixed PA/MR model.

Results for the analyses for surgical specialties only are described in S2 Table. The patients on the wards with a PA/MD model had a significantly higher incidence of pressure ulcer (OR 0.42, 95% CI 0.21–0.88) and episode of at least two days pain score ≥ 7 (OR 0.21, 95% CI 0.15–0.67), but a significantly lower number of presentations at the department of emergency after discharge (OR 1.47, 95% CI 1.02–2.13). Evaluations of patients were significantly better on wards with the PA/MD model.

In S3 Table the results for the non-surgical wards are summarized. We found significant differences in the incidence of presentation at the department of emergency and unplanned readmission in favor of the control group. The number of days between discharge and discharge letter differed significantly in favor of the intervention group: β -0.22, 95% -1.00–0.57.

## Discussion

In the present study, we aimed to determine the effects of substitution of inpatient care from MDs to PAs on patients’ LOS, quality and safety of care, and patient experiences with care provided. No difference between the two study arms was found on these measures, except that the involvement of PAs was significantly associated with better patient experiences. In particular, patients rated communication, continuity, cooperation and medical care better on wards with PAs.

Our findings do not confirm our hypothesis that patients’ LOS would be shorter on wards on which PAs are involved in inpatient care. Reducing LOS is an aim for policy makers in many health care systems [21]. As a consequence, in the Netherlands as well as in many other countries, reducing LOS has been of major interest in the previous decade [22]. Due to several interventions, the average LOS decreased from 11.2 days in 1990 to 9.0 days in 2000 and 6.4 days in 2012 [23]. Although there are still variations in LOS between countries and hospitals, it is debatably what decrease of LOS is feasible.

To our knowledge, this is the first multicenter study that investigates the effects of reallocating inpatient care from MDs to PAs. A few single-centered studies have compared non-acute inpatient care delivered by a PA-based team with the care delivered by a resident-based team [9–13]. All studies reported similar quality of care for PA and non-PA care, which is in line with our results. However, the results regarding LOS were mixed. Singh et al [10] reported that the PA-based team was associated with an increased patients’ LOS, while Nishimura et al [12] and Miller et al [13] reported an association with a decreased LOS. Comparable to our results, Roy et al [9]and Dupher et al [11] showed similar LOS between de study arms. These studies can however hardly be compared, because different methodology was used and different patient groups were involved. Besides, most of these studies compared a hospitalist/PA model with the traditional resident-based model, while hospitalists were not part of the models we involved [15]. Hospitalists have been introduced in the Netherlands since 2012 and were not graduated yet at the start of our study. The PAs in our intervention model were supervised by staff physicians of the specific clinical discipline, instead of the hospitalists who have a supervising role in the PA/hospitalist models in the USA. Based on the descriptions, the tasks of the PAs who are employed for inpatient care in the Netherlands, appear to be largely comparable to the tasks of the PAs in the USA, which makes it unlikely that differences in team composition would affect the results.

Contrary to some of above mentioned studies which showed no differences between PAs and MDs on patient experiences [9, 11, 12], we found significantly better patient experiences on wards with PAs. This difference in findings might be the result of a specific focus on experiences in medical inpatient care, whereas the other studies focused on the general care-giving team with often low response rates. Nonetheless, one could debate about the relevance of the statistically significant differences on patient experiences, since the scores in both groups indicate (very) positive experiences.

Although the study was not designed to confirm equivalence between study arms, our study suggests that the care on wards with the PA/MD model is not different from the care on the wards with traditional house staffing. Employing a PA for inpatient care seems to be safe. PAs may be a cost-effective alternative for residents and hospitalists, because they can be trained faster and the cost of their training is significantly lower compared to MDs. As shown in Table 2, the time spend on inpatient care (i.e. workload at the ward) is less in the PA/MD group than in the MD group. This indicates advantages on health care costs as well. The less time might be related to our previous finding that the provider continuity is more constant on wards with PAs, and that PAs are more experienced than residents [15]. As a consequence, PAs might be more familiar with the clinical protocols and the procedures to for example request diagnostics tests and consultation of other (sub)specialties. Therefore, they spend less time on such indirect patient care. Furthermore, as a consequence of the higher provider continuity, PAs might be more familiar with the routines of other individual professionals, the medical team on the ward and multidisciplinary teams [15].

A strength of this study is the multicenter design and high response rate on all three patient questionnaires, which enhances the representativeness of our findings. Besides, we were able to include a broad range of clinical disciplines from different types of hospitals, which increases the generalizibility of our findings. We included 15 wards in teaching hospitals and 19 wards in non-teaching hospitals. This is approximately in proportion with the Dutch situation; 36 teaching hospitals and 60 nonteaching hospitals [24]. Although we have not selectively recruited the wards, most of the included wards were from a surgical (sub)specialty. There are no exact data about the number of PAs who are employed specifically for the management of hospitalized patients per clinical discipline, but we know that in the Netherlands most of them are employed at a surgical department. Some clinical disciplines, like internal medicine and obstetrics/gynecology, were however not represented at all. It is not clear whether our results can be extrapolated to those disciplines. Besides, it is not clear whether the results can be generalized to other countries where PAs have more mobility between setting and specialties compared to the Netherlands.

A limitation is the non-randomized design of this study. Different from other countries, the Dutch PA programs incorporate a dual work-education model, which means that students are employed within a particular medical specialty from the day of their enrollment in the master’s PA program [25, 26]. After graduation, the majority of PAs continue their employed at the same department. The suggestion of randomly reallocating the graduated PA to other hospital wards was considered infeasible for the staff physicians, who put considerable effort and time in training and supervision. The non-randomized character of this study implies an increased risk for confounding, which we took into account in the multivariable analyses. However, we cannot exclude that local differences like policies about quality of care and patient case-mix could have influenced our results. To explore heterogeneity within our data, we conducted subgroup analyses for the four organizational models for medical ward care separately. Although the results of subgroup analyses should be interpreted with caution because of low numbers of patients per subgroup, several findings are intriguing. Significant differences in favor of the model in which only medical specialists were involved were found regarding the indicators the incidence of hospital infections, pressure ulcer, episode of two days body temperature ≥38, and episodes of two days Numeric Rating Score ≥7. This might indicate higher quality of care within this model. We cannot exclude that this indicates that the patients which were included in this model were overall less complex than the patients in the other models. Although we’ve adjusted for relevant confounders in the multivariable analysis, it is not possible to perfectly adjust for the complexity of the patient. Further research should explore the cause of the difference.

We also performed separate analyses for surgical specialties only and non-surgical specialties only.

We found significant differences for some indicators for quality and safety of care that were not consistent in favor of one of the study arms. Remarkably, the difference in patient evaluations between the study arms remained for the subgroup with surgical specialties, but not for the subgroup with non-surgical specialties. Reasons remain however speculative.

## Conclusion

This study did not find differences regarding LOS and quality of care between wards on which PAs, in collaboration with MDs, provided medical care for the admitted patients, and wards on which only MDs provide medical care. Employing PAs seems to be safe and seems to lead to better patient experiences.

## Supporting information

S1 Table Results per submodel of medical ward care. (DOCX)

S2 Table Results for surgical (sub)specialties only. (DOCX)

S3 Table Results for non-surgical specialties only. (DOCX)

S1 File Used questions_Dutch. (DOCX)

S2 File Used questions_English. (DOCX)
