# Supplementary material for: The impact of the implementation of physician assistants in inpatient care: A multicenter matched-controlled study
Source: PLoS One. 2017 Aug 9;12(8):e0178212. doi: 10.1371/journal.pone.0178212 (PMC5549960; doi:10.1371/journal.pone.0178212)
Supplement: S1 Table — (DOCX) [file pone.0178212.s001.docx]

**Table S1. Results per submodel of medical ward care**

| **Outcome** |  | **PA/MD model** | |  | **MD model** | |  | ***P*-value^c,d^** |
| --- | --- | --- | --- | --- | --- | --- | --- | --- |
|  |  | **PA/MR model**  **(n=686)** | **PA model**  **(n=309)** |  | **MR model**  **(n=908)** | **MS model**  **(n= 345)** |  |  |
| **Length of hospital stay** *median (IQR)* ^a^ |  | 7 (4-11) | 5 (3-8) |  | 6 (4-9) | 4 (3-6) |  | NS |
|  |  |  |  |  |  |  |  |  |
| **Indicators for quality of care** |  |  |  |  |  |  |  |  |
| In-hospital mortality  *n(%)* |  | 2/683 (0.3%) | 0/308 (0%) |  | 0/906 (0%) | 1/325 (0.3%) |  | NA |
| Unplanned transfer to ICU  *n(%)* |  | 15/681 (2%) | 4/306 (1%) |  | 23/896 (3%) | 1/344 (0.3%) |  | NS |
| Cardiopulmonary resuscitation  *n(%)* |  | 1/681 (0.1%) | 0/307 (0%) |  | 1/903 (0.1%) | 0/352 (0%) |  | NA |
| Pressure ulcer developed during admission  *n(%)* |  | 16/600 (3%) | 15/289 (5%) |  | 10/815 (1%) | 0/0 (0%) |  | 2 vs 4: *P*=0.045 |
| Episode of at least 2 days temp ≥38  *n(%)* |  | 160/677 (24%) | 93/298 (31%) |  | 254/885 (29%) | 41/344 (12%) |  | 1 vs 2: *P*=0.003  2 vs 4: *P*=0.000  2 vs 3: *P*=0.002  3 vs 4: *P*=0.023 |
| Episode of at least 2 days pain score ≥7  *n(%)* |  | 44/671 (7%) | 13/308 (4%) |  | 31/878 (4%) | 3/285 (1%) |  | 1 vs 3:  *P*=0.046  1 vs 4:  *P*=0.023 |
| Hospital infection^b^ *n(%)* |  | 51/675 (8%) | 11/304 (4%) |  | 63/891 (7%) | 2/320 (1%) |  | 3 vs 4: *P*=0.041 |
| Presentation at department of emergency  *n(%)* |  | 59/564 (10%) | 22/248 (9%) |  | 95/733 (13%) | 25/249 (10%) |  | NS |
| Unplanned readmission  *n(%)* |  | 55/497 (11%) | 12/237 (5%) |  | 59/652 (9%) | 18/277 (7%) |  | NS |
| Introduction to patient <24h  *n(%)* |  | 469/562 (84%) | 185/245 (76%) |  | 633/730 (87%) | 185/246 (75%) |  | NS |
| Days between discharge and discharge letter  *median (IQR)* |  | 0 (0-6) | 3 (0-10) |  | 5 (0-14) | 4 (0-19) |  | NS |
| **Patient satisfaction** |  |  |  |  |  |  |  |  |
| Overall satisfaction score *mean (SD)* |  | 8.4 (1.2) | 8.4 (1.3) |  | 8.0 (1.5) | 8.1 (1.7) |  | 1 vs 3: *P*=0.013  2 vs 3: *P*=0.009  2 vs 4: *P*=0.034 |
| Communication  *mean (SD)* |  | 4.1 (0.7) | 4.2 (0.7) |  | 4.0 (0.8) | 4.0 (0.9) |  | 1 vs 3:  *P*=0.034  2 vs 3:  *P*=0.010 |
| Continuity  *mean (SD)* |  | 4.7 (1.1) | 4.7 (1.1) |  | 4.4 (1.2) | 4.4 (1.3) |  | 1 vs 3:  *P*=0.017  2 vs 3:  *P*=0.020 |
| Cooperation  *mean (SD)* |  | 4.7 (1.1) | 4.8 (1.1) |  | 4.4 (1.2) | 4.4 (1.3) |  | 1 vs 3:  *P*=0.041  2 vs 3:  *P*=0.021 |
| Medical care  *mean (SD)* |  | 4.8 (1.0) | 4.9 (1.1) |  | 4.6 (1.1) | 4.7 (1.2) |  | NS |

Abbreviations: NA=not applicable because of limited number of cases; IQR=interquartile range

a. log-transformed before regression analysis

b. i.e. Infusion, urinary track, airway and/or postoperative wound infection

c. Adjusted for medical specialty, hospital type and type of admission

d. 1= Mixed PA/MD model; 2= PA model; 3=MR model; 4=MS model
