# Supplementary material for: The impact of the implementation of physician assistants in inpatient care: A multicenter matched-controlled study
Source: PLoS One. 2017 Aug 9;12(8):e0178212. doi: 10.1371/journal.pone.0178212 (PMC5549960; doi:10.1371/journal.pone.0178212)
Supplement: S2 Table — (DOCX) [file pone.0178212.s002.docx]

**Table S2. Results for surgical (sub)specialties only**

Abbreviations: NA=not applicable because of limited number of cases; IQR=interquartile range

| **Outcome** | **PA/MD model** | **MD model** | **Estimates** | |
| --- | --- | --- | --- | --- |
|  | **(n=709)** | **(n=856)** | **β ^c^** | **95% CI** |
| **Length of hospital stay** *median (IQR) ^a^* | 6 (4-9) | 5 (3-8) | 0.26 | -0.03-0.55 |
|  |  |  |  |  |
| **Indicators for quality of care** |  |  | **OR ^c^** | **95% CI** |
| In-hospital mortality *n(%)* | 1/705 (0.1%) | 0/855 (0.0%) | NA | NA |
| Unplanned transfer to ICU  *n(%)* | 41/704 (6%) | 68/853 (8%) | 1.03 | 0.58-1.82 |
| Cardiopulmonary resuscitation  *n(%)* | 0/702 (0.0 %) | 1/853 (0.1%) | NA | NA |
| Pressure ulcer developed during admission *n(%)* | 27/624 (4%) | 10/737 (1%) | 0.43** | 0.21-0.88 |
| Episode of at least 2 days temp ≥38  *n(%)* | 191/694 (28%) | 242/856 (28%) | 0.90 | 0.69-1.15 |
| Episode of at least 2 days pain score ≥7  *n(%)* | 30/701 (4%) | 13/846 (2%) | 0.32** | 0.15-0.67 |
| Hospital infection^b^ *n(%)* | 36/696 (5%) | 53/842 (6%) | 1.10 | 0.63-1.81 |
| Presentation at department of emergency  *n(%)* | 79/537 (15%) | 121/654 (19%) | 1.47** | 1.02-2.13 |
| Unplanned readmission  *n(%)* | 42/543 (8%) | 48/655 (7%) | 1.00 | 0.59-1.70 |
| Introduction to patient <24h  *n(%)* | 431/558 (77%) | 513/622(82%) | 1.42 ** | 1.01-2.01 |
| **Indicators for quality of care** |  |  | **β ^c^** | **95% CI** |
| Days between discharge and discharge letter  *median (IQR) ^a^* | 2 (0-9) | 4 (0-17) | -0.22 | -1.00-0.57 |
|  |  |  |  |  |
| **Patient satisfaction** |  |  | **β ^c^** | **95% CI** |
| Overall satisfaction score *mean (SD)* | 8.40 (1.27) | 7.83 (1.58) | 0.66** | 0.35-0.97 |
| Communication  *mean (SD)* | 4.17 (0.72) | 3.89 (0.82) | 0.36** | 0.17-0.55 |
| Continuity  *mean (SD)* | 4.69 (1.13) | 4.27 (1.24) | 0.45** | 0.19-0.71 |
| Cooperation  *mean (SD)* | 4.78 (1.07) | 4.30 (1.24) | 0.49** | 0.24-0.75 |
| Medical care  *mean (SD)* | 4.87 (1.05) | 4.52 (1.18) | 0.40** | 0.13-0.68 |

a. log-transformed before regression analysis

b. i.e. Infusion, urinary track, airway and/or postoperative wound infection

c. Adjusted for medical specialty, hospital type, primary diagnosis, type of admission and discharge destination

** *P* < .05
