# Supplementary material for: The impact of the implementation of physician assistants in inpatient care: A multicenter matched-controlled study
Source: PLoS One. 2017 Aug 9;12(8):e0178212. doi: 10.1371/journal.pone.0178212 (PMC5549960; doi:10.1371/journal.pone.0178212)
Supplement: S3 Table — (DOCX) [file pone.0178212.s003.docx]

**Table S3. Results for non-surgical specialties only**

| **Outcome** | **PA/MD model** | **MD model** | **Estimates** | |
| --- | --- | --- | --- | --- |
|  | **(n=291)** | **(n=410)** | **β ^b^** | **95% CI** |
| **Length of hospital stay** *median (IQR) ^a^* | 7 (5-11) | 6 (4-8) | 0.14 | -0.20-0.48 |
|  |  |  |  |  |
| **Indicators for quality of care** |  |  | **OR ^b^** | **95% CI** |
| In-hospital mortality *n(%)* | 1/286 (0.3%) | 1/376 (0.3%) | NA | NA |
| Unplanned transfer to ICU  *n(%)* | 5/286 (2%) | 2/396 (1%) | 0.55 | 0.17-1.74 |
| Cardiopulmonary resuscitation  *n(%)* | 1/286 (0.3%) | 0/374 (0%) | NA | NA |
| Pressure ulcer developed during admission *n(%)* | 4/265 (2%) | 0/410 (0%) | 0.74 | 0.17-3.15 |
| Episode of at least 2 days temp ≥38  *n(%)* | 62/285 (22%) | 67/393 (17%) | 0.61 | 0.35-1.05 |
| Episode of at least 2 days pain score ≥7  *n(%)* | 27/282 (10%) | 21/336 (6%) | 0.43 | 0.18-1.05 |
| Hospital infection ^c^ *n(%)* | 26/283 (9%) | 12/369 (3%) | 0.26 | 0.06-1.03 |
| Presentation at department of emergency  *n(%)* | 39/202 (19%) | 47/281 (17%) | 0.22** | 0.08-0.58 |
| Unplanned readmission  *n(%)* | 25/191 (13%) | 29/274 (11%) | 0.20** | 0.06-0.62 |
| Introduction to patient <24h  *n(%)* | 223/249 (90%) | 305/354 (86%) | 1.65 | 0.31-1.36 |
| **Indicators for quality of care** |  |  | **β ^b^** | **95% CI** |
| Days between discharge and discharge letter  *median (IQR) ^a^* | 0 (0-2) | 4 (1-10) | -1.35** | -2.56- -0.13 |
|  |  |  |  |  |
| **Patient satisfaction** |  |  | **β ^b^** | **95% CI** |
| Overall satisfaction score *mean (SD)* | 8.43 (1.22) | 8.30 (1.45) | 0.56 | -0.33-1.45 |
| Communication  *mean (SD)* | 4.13 (0.73) | 4.09 (0.76) | 0.20 | -0.22-0.62 |
| Continuity  *mean (SD)* | 4.59 (1.11) | 4.55 (1.17) | 0.26 | -0.67-1.20 |
| Cooperation  *mean (SD)* | 4.49 (1.19) | 4.58 (1.21) | 0.14 | -0.68-0.95 |
| Medical care  *mean (SD)* | 4.74 (0.97) | 4.75 (1.07) | 0.26 | -0.55-1.06 |

Abbreviations: NA=not applicable because of limited number of cases; IQR=interquartile range

a. log-transformed before regression analysis

b. i.e. Infusion, urinary track, airway and/or postoperative wound infection

c. Adjusted for medical specialty, hospital type, primary diagnosis, type of admission and discharge destination

** *P* < .05
