# Supplementary material for: The impact of the implementation of physician assistants in inpatient care: A multicenter matched-controlled study
Source: PLoS One. 2017 Aug 9;12(8):e0178212. doi: 10.1371/journal.pone.0178212 (PMC5549960; doi:10.1371/journal.pone.0178212)
Supplement: S1 File — (DOCX) [file pone.0178212.s004.docx]

|  | **De zaalarts..** | | | | | **Slecht** | **Matig** | **Goed** | **Zeer goed** | **Uitstekend** | **N.v.t.** |  |
| --- | --- | --- | --- | --- | --- | --- | --- | --- | --- | --- | --- | --- |
| 1. | … Begroette mij op een manier waardoor ik een  comfortabel gevoel kreeg | | | | | O | O | O | O | O | O |  |
| 2. | … Behandelde mij met respect | | | | | O | O | O | O | O | O |  |
| 3. | … Toonde interesse in mijn ideeën over mijn  gezondheid | | | | | O | O | O | O | O | O |  |
| 4. | … Begreep mijn belangrijkste  gezondheidsklachten | | | | | O | O | O | O | O | O |  |
| 5. | … Schonk mij aandacht (keek mij aan, luisterde  aandachtig) | | | | | O | O | O | O | O | O |  |
| 6. | … Liet mij uitpraten zonder onderbrekingen | | | | | O | O | O | O | O | O |  |
| 7. | … Gaf mij zoveel informatie als ik wilde | | | | | O | O | O | O | O | O |  |
| 8. | … Sprak in termen die ik kon begrijpen | | | | | O | O | O | O | O | O |  |
| 9. | … Ging na of ik alles begreep | | | | | O | O | O | O | O | O |  |
| 10. | … Moedigde mij aan vragen te stellen | | | | | O | O | O | O | O | O |  |
| 11. | … Betrok mij in beslissingen zoveel als ik wilde | | | | | O | O | O | O | O | O |  |
| 12. | … Besprak de volgende stappen | | | | | O | O | O | O | O | O |  |
| 13. | … Toonde zorg en aandacht | | | | | O | O | O | O | O | O |  |
| 14. | … Besteedde de juiste hoeveelheid tijd aan mij | | | | | O | O | O | O | O | O |  |
|  |  | | | | |  |  |  |  |  |  |  |
|  | **De zaalarts..** | | | | | **Onvol-doende** | **Twijfel-achtig** | **Vol-doende** | **Ruim vol-doende** | **Goed** | **Zeer goed** | **N.v.t.** |
| 15. | … Weet welke symptomen al eerder zijn  besproken | | | | | O | O | O | O | O | O | O |
| 16. | … Weet wat de andere zorgverlener heeft  gedaan/ wat de behandeling is | | | | | O | O | O | O | O | O | O |
|  | **Als u terugkijkt naar deze opname, wat vindt u dan van de zaalarts als het gaat om…** | | | | |  |  |  |  |  |  |  |
| 17. | … Het snel verlichten van uw klachten? | | | | | O | O | O | O | O | O | O |
| 18. | … Een zorgvuldige en degelijke aanpak? | | | | | O | O | O | O | O | O | O |
| 19. | … Lichamelijk onderzoek doen bij u | | | | | O | O | O | O | O | O | O |
|  |  | | | | |  |  |  |  |  |  |  |
| 20. | **Kunt u op een schaal van 1 tot 10 aangeven hoe tevreden u bent over de zaalarts in het algemeen?** | | | | | | | | | | | |
|  |  |  | **Slecht** |  |  |  | **Voldoende** | | |  | **Uitstekend** | |
|  |  |  | O | O | O | O | O | O | O | O | O | O |
|  |  |  | **1** | **2** | **3** | **4** | **5** | **6** | **7** | **8** | **9** | **10** |

**Questions used for measuring patients’ experiences**

**Questions used for measuring readmission and presentation at emergency department within one month after discharge**

1. Hoe vaak bent u sinds uw ontslag op een EHBO of spoedeisende hulp geweest voor uzelf?

- 0 keer -> ga door naar vraag 4
- 1 keer
- 2 keer
- Vaker, namelijk … keer

1. Had een van deze bezoeken aan de EHBO of spoedeisende hulp te maken met hetzelfde probleem als waarvoor u ruim 1 maand geleden was opgenomen in het ziekenhuis?

- Ja
- Nee -> ga door naar vraag 4

1. Hoeveel van deze bezoeken aan de EHBO of spoedeisende hulp hadden te maken met hetzelfde probleem als waarvoor u ruim 1 maand geleden was opgenomen in het ziekenhuis?

- 1 bezoek
- 2 bezoeken
- 3 bezoeken
- Anders, namelijk …. bezoeken

1. Bent u sinds uw ontslag uit het ziekenhuis van ongeveer 1 maand geleden opnieuw opgenomen geweest in een willekeurig ziekenhuis?

- Ja
- Nee -> ga door naar vraag 6

1. Wilt u alstublieft in onderstaande tabel voor iedere nieuwe opname sinds uw ontslag van 1 maand geleden het volgende aangeven: De opnamedatum, de ontslagdatum, of deze opname te maken had met de opname van 1 maand geleden, of deze opname gepland was, en op welke afdeling (medisch specialisme) u opgenomen was.

| **Opnamedatum**  dag-maand-jaar | **Ontslagdatum**  dag-maand-jaar | **Gerelateerd aan opname van 1 maand geleden?** | **Geplande opname?*** | **Medisch specialisme** |
| --- | --- | --- | --- | --- |
| ……. - ……. - ………. | ……. - ……. - ………. | - Ja - Nee | - Ja - Nee | …………………….… |
| ……. - ……. - ………. | ……. - ……. - ………. | - Ja - Nee | - Ja - Nee | …………………….… |
| ……. - ……. - ………. | ……. - ……. - ………. | - Ja - Nee | - Ja - Nee | ………………………. |

*geplande opname = datum stond vantevoren vast
