# Supplementary material for: The impact of the implementation of physician assistants in inpatient care: A multicenter matched-controlled study
Source: PLoS One. 2017 Aug 9;12(8):e0178212. doi: 10.1371/journal.pone.0178212 (PMC5549960; doi:10.1371/journal.pone.0178212)
Supplement: S2 File — (DOCX) [file pone.0178212.s005.docx]

|  | **The medical care provider at the ward..** | | | | | **Poor** | **Fair** | **Good** | **Very good** | **Excellent** | **NA** |  |
| --- | --- | --- | --- | --- | --- | --- | --- | --- | --- | --- | --- | --- |
| 1. | … Greeted me in a way that made me feel  comfortable | | | | | O | O | O | O | O | O |  |
| 2. | … Treated me with respect | | | | | O | O | O | O | O | O |  |
| 3. | … Showed interest in my ideas about my health | | | | | O | O | O | O | O | O |  |
| 4. | … Understood my main health concerns | | | | | O | O | O | O | O | O |  |
| 5. | … Paid attention to me (looked at me, listened) | | | | | O | O | O | O | O | O |  |
| 6. | … Let me talk without interruptions | | | | | O | O | O | O | O | O |  |
| 7. | … Gave me as much information as I wanted | | | | | O | O | O | O | O | O |  |
| 8. | … Talked in terms I could understand | | | | | O | O | O | O | O | O |  |
| 9. | … Checked to be sure I understood everything | | | | | O | O | O | O | O | O |  |
| 10. | … Encouraged me to ask questions | | | | | O | O | O | O | O | O |  |
| 11. | … Involved me in decisions as much as I wanted | | | | | O | O | O | O | O | O |  |
| 12. | … Discussed next steps | | | | | O | O | O | O | O | O |  |
| 13. | … Showed care and concern | | | | | O | O | O | O | O | O |  |
| 14. | … Spent the right amount of time with me | | | | | O | O | O | O | O | O |  |
|  |  | | | | |  |  |  |  |  |  |  |
|  | **The medical care provider at the ward..** | | | | | **Poor** | **Fair** | **Sufficient** | **Amply sufficient** | **Good** | **Excellent** | **NA** |
| 15. | … Knows which symptoms have been discussed  before | | | | | O | O | O | O | O | O | O |
| 16. | … Knows what other provider has  done/what the treatment is | | | | | O | O | O | O | O | O | O |
|  | **When you look back to this admission, what do you think about the medical care provider with respect to…** | | | | |  |  |  |  |  |  |  |
| 17. | … quickly relievement of complaints | | | | | O | O | O | O | O | O | O |
| 18. | … a carefull and thorough approach | | | | | O | O | O | O | O | O | O |
| 19. | … performing physical examination | | | | | O | O | O | O | O | O | O |
|  |  | | | | |  |  |  |  |  |  |  |
| 20. | **On a scale from 1 to 10, how satisfied are you in general with the medical care provider at the ward?** | | | | | | | | | | | |
|  |  |  | **Poor** |  |  |  | **Sufficient** | | |  | **Excellent** | |
|  |  |  | O | O | O | O | O | O | O | O | O | O |
|  |  |  | **1** | **2** | **3** | **4** | **5** | **6** | **7** | **8** | **9** | **10** |

**Questions used for measuring patients’ experiences**

**Questions used for measuring readmission and presentation at emergency department within one month after discharge**

1. How often have you visited the emergency department since your discharge about one month ago?

- Never -> go to question 4
- 1 time
- 2 times
- More often, namely ….. times

1. Was one of those visits at the emergency department related to the problem for which you were admitted to the hospital about one month ago?

- Yes
- No -> go to question 4

1. How many of those visits to the emergency department were related to the problem for which you were admitted to the hospital about one month ago?

- 1 visit
- 2 visits
- 3 visits
- Other, namely ..… visits

1. How you been readmitted to any hospital since your discharge about one month ago?

- Yes
- No -> go to question 6

1. Please mention the following items about your readmission(s) in the table below: date of admission, date of discharge, whether the readmission was related to the initial admission, whether the readmission was elective, and for which medical specialty you were readmitted

| **Date of admission**  day-month-year | **Date of discharge**  day-month-year | **Related to the initital admission 1 month ago?** | **Elective readmission?*** | **Medical specialty** |
| --- | --- | --- | --- | --- |
| ……. - ……. - ………. | ……. - ……. - ………. | - Yes - No | - Yes - No | …………………….… |
| ……. - ……. - ………. | ……. - ……. - ………. | - Yes - No | - Yes - No | …………………….… |
| ……. - ……. - ………. | ……. - ……. - ………. | - Yes - No | - Yes - No | ………………………. |

*elective readmission = date was scheduled
